# Pain and recurrent falls in the older and oldest-old non-institutionalized population

**DOI:** 10.1186/s12877-020-1412-8

**Published:** 2020-01-14

**Authors:** César Gálvez-Barrón, Francesc Formiga, Antonio Miñarro, Oscar Macho, Leire Narvaiza, María Dolores Dapena, Ramon Pujol, Alejandro Rodríguez-Molinero

**Affiliations:** 1 Clinical Research Unit and Department of Geriatrics, Consorci Sanitari de l’Alt Penedès i Garraf, Ronda Sant Camil s/n, Sant Pere de Ribes, 08810 Barcelona, Spain; 2 Geriatric Unit. Department of Internal Medicine, IDIBELL, Universitat de Barcelona, Hospital Universitari de Bellvitge, Barcelona, Spain; 3 Department of Genetics, Microbiology and Statistics, School of Biology, Universitat de Barcelona, Barcelona, Spain; 4 Department of Geriatrics, Consorci Sanitari de l’Alt Penedès i Garraf, Barcelona, Spain; 5 Psychogeriatric Unit, Hospital Benito Menni, Barcelona, Spain; 6 School of Medicine, Universitat de Vic-Universitat Central de Catalunya, Barcelona, Spain

**Keywords:** Oldest-old people, Falls, Risk factors

## Abstract

**Background:**

Recurrent falls represent a priority in geriatric research. In this study we evaluated the influence of pain as a risk factor for recurrent falls (two or more in 1 year) in the older (65–79 years) and oldest-old (80 or more years) non-institutionalized population.

**Methods:**

Prospective cohort study. 772 non-institutionalized individuals with ages of 65 years or older (with overrepresentation of people aged 80 years or older [*n* = 550]) were included through randomized and multistage sampling, stratified according to gender, geographic area and habitat size. Basal evaluation at participant’s home including pain evaluation by Face Pain Scale (FPS, range 0–6) and then telephonic contact every 3 months were performed until complete 12 months. Multivariate analysis by logistic regression (recurrent falls as outcome variable) for each age group (older and oldest-old group) were developed considering pain as a quantitative variable (according to FPS score). Models were adjusted for age, gender, balance, muscle strength, depressive symptoms, cognitive decline, number of drugs and number of drugs with risk of falls.

**Results:**

114 (51.35%) and 286 (52%) participants of older and oldest-old group, respectively, reported pain; and recurrent falls occurred in 6.93% (*n* = 12) of the older group and 12.06% (*n* = 51) of the oldest-old group. In the older group, pain was associated with recurrent falls, with an associated odds ratio (OR) of 1.47 (95% CI 1.08–2.00; beta 0.3864) for each unit increase in pain intensity (thus, participants with the most severe pain [FPS 6] had OR of 10.16 regarding to participants without pain [FPS 0]). In the oldest-old group, pain was not associated with recurrent falls.

**Conclusions:**

Pain, a potentially modifiable and highly prevalent symptom, is a risk factor for recurrent falls in the older people (65–79 years). However, we have not been able to demonstrate that this relationship is maintained in the oldest-old population (80 or more years).

## Background

Falls and their sequelae represent a major problem in the older population because they are associated with various adverse events, such as fractures, functional deterioration, institutionalization, and visits to emergency services [1–4]. In relation to the number of falls, the profile of older individuals with only one fall (in 6–12 months) is more similar to that of older individuals who do not fall than to that of those who have recurrent falls (in terms of visual acuity, reaction time, body sway, quadriceps strength, and other parameters); therefore, knowing the characteristics of patients with recurrent falls is especially relevant [5].

When evaluating falls, the evaluation and treatment of the identified modifiable risk factors are very important [6, 7]. However, the net benefit of this approach is considered small at the population scale [7]; therefore, the identification of new risk factors that are still not known or not confirmed represents a care priority. In this sense, pain as a risk factor for recurrent falls is relevant because it is a highly prevalent (25–75% of the older population) [8] and potentially modifiable factor. While there are studies that evaluate this relationship in the older population, few studies are based on prospective primary data. Thus, the meta-analysis by Stubbs et al. [9] included 3 prospective studies [10–12], of which only the work by Leveille et al. [10] evaluated this relationship in a primary way in an exclusively female population with functional disability. Recently, a positive association was reported in an autonomous male population (unassisted ambulation) [13], as well as a positive relationship for lumbar pain specifically [14, 15].

Additionally, the aforementioned studies do not distinguish the older population (65–79 years) from the oldest-old population (80 years or older). This distinction is increasingly relevant because the first group, given the favourable impact of medicine and the consequent increase in life expectancy, tends to maintain a higher level of autonomy and physical activity [16, 17]. This difference could influence the risk of falling and its association with pain, making it difficult to extrapolate results between the 2 groups. In a meta-analysis [9], the influence of age on the relationship of pain with recurrent falls could not be evaluated.

Given the above, the objective of this study is to prospectively evaluate the influence of pain as a risk factor for recurrent falls in the older and oldest-old non-institutionalized population.

## Methods

### Design and sample

This is an observational prospective cohort study. The cohort has been previously described [18]. In summary and in relation to this study, 772 non-institutionalized individuals with ages of 65 years or older were included; the participants were residents of all the provinces of Spain and were recruited from the last available population census through randomized, multistage sampling, stratified according to gender, geographic area and habitat size. Sampling was unproportionally allocated with overrepresentation of people aged 80 years or older (*n* = 550). To minimize the lack of response from potential participants, only one individual candidate was substituted for another in case of 10 failed contact attempts, 2 failed scheduled appointments, negativity towards participation or inability to participate, institutionalization or death. Regarding the contact method, within each population, neighbourhoods or districts were selected, and within the district, households were selected through a mixed system of door-to-door sampling and telephone contact. Primary research topics included pain, falls, vital signs, and assessment of gait and balance. The recruitment and follow-up period of the cohort was performed during the years 2007–2009.

Considering an alpha risk of 0.05 and a beta risk of 0.2 in a bilateral contrast, a relative risk (RR) of 1.75, a pain prevalence of 50% and of recurrent falls between participants without pain of 15%, a Poisson approximation, and a loss rate of 10%, the number of participants needed for this study was 488.

### Data collection

A face-to-face baseline assessment was performed at the home of the participant and then follow-up telephone contact at 4, 6, 9 and 12 months. The basal data were collected by professional surveyors, all trained, via theoretical and practical sessions, by the study researchers using the same method. Follow-up data were collected through telephone interviews conducted by personnel of the centre responsible for the study (different from the interviewers who performed the baseline assessment) using a structured interview model for which they received the same theoretical and practical training. The individuals responsible for the follow-up calls were not aware of the hypothesis of this study, and although they had access to the data from the baseline assessment, knowledge of the data was not necessary for the follow-up calls.

### Pain-related variables

The presence of pain was evaluated verbally in the baseline interview through the following question: “Have you had pain in any part of the body in the last 4 weeks?”. In the case of an affirmative answer, the following question was asked: “where?”. The interviewer copied the response given by the participant literally, and all body areas reported were recorded.

For the participants who responded to having pain, its intensity was evaluated through the application of the Face Pain Scale (FPS) [19], which has a scale comprehension verification question; therefore, if the participant did not comprehend the scale, pain intensity was not assessed. If more than one affected body area was reported, the area with the highest pain intensity was considered. The FPS is a self-report scale (type of scale recommended for pain assessment [20]) that has shown validity in the older population [21]. It has also demonstrated its usefulness in patients suffering from moderate-severe dementia who maintain communication capacity [22].

### Fall-related variables

A fall was defined as any event by which the person ends up on the floor, or in a lower plane, in an unintentional way. However, during contact with the participant, the colloquial term “fall” was used and included in direct and simple questions (“Have you fallen since the last call?”), without giving the operative definition to the participant. As reported by the participant, the pollster decided whether a fall had occurred.

During each telephone follow-up contact, the participant (or close informant) was asked about the occurrence of falls with respect to the baseline assessment or the last telephone contact made. Participants with 2 or more falls during follow-up were considered individuals with recurrent falls, and participants were considered to have completed the follow-up if fall data were available for 12 months.

### Other variables

In the baseline assessment, the functional status, cognitive decline, and the presence of affective symptoms and comorbidities were evaluated through the Katz index [23] (range 0 to 6, where 6 represents a dependent person for all basic activities of daily living), the Pfeiffer test [24] (version adapted to Castilian Spanish by Martínez De La Iglesia et al. [25], range 0–10, cut-off point ≥3), the 5-question Yesavage Geriatric Depression Scale (GDS-5) [26] (version adapted to Spanish by Ortega Orcos et al. [26], range 0–5, cut-off point ≥2), and the list of chronic diseases included in the questionnaire of the Spanish National Health Survey [27]; respectively. In addition, visual acuity was also assessed (according to the ability or not to recognize another person at a distance of 4 m at the other side of the street with or without the aid of glasses).

The strength of the lower limbs (foot, knee and hip) was measured by manual measurement through the Medical Research Council scale [28], whose values range from 0 to 5 for each muscle group, with 5 being normal strength. Balance was assessed through observation of the participant in a seated position, of the ability of the participant to get up without help, of the balance of the participant in the standing position after standing up (5 s) and of the participant standing, according to the 4 corresponding sections of the Tinetti scale [29] and using the same scoring system (0–1 for the sitting balance section and 0–2 for the other three parameters). Lastly, pharmacological treatments were recorded (participants were asked to show the interviewer all the medications they took regardless of whether the medications were prescribed by a doctor) and the use of technical aids to walk.

Before disposing the results, total strength, balance, and affective symptoms were considered potential confounders. We did not consider osteoarthritis as a potential confounder because a poor correlation has been demonstrated between self-reported diagnosis (the method of this study) and radiological confirmation of this pathology (it has been proposed that self-reported diagnosis is more an indication of joint pain than of the presence of this pathology) [10, 30]. Additionally, it has not been fully demonstrated that osteoarthritis is a risk factor for falls and could even be a protective factor [31].

### Statistical analysis

For all analyses, the sample was divided into 2 age groups: 65–79 years (older age group) and 80 years or older (oldest-old group). No other subgroups were planned.

Pain and its intensity were grouped into the following categories: no pain (FPS 0), mild pain (FPS 1–2), moderate pain (FPS 3–4) and severe pain (FPS 5–6). For the analysis of the balance variables and Katz index, the total scores of the respective applied measurement tools (ranges 0–7 and 0–6, respectively) were considered. In the case of strength, the average of the right and left scores of each joint was calculated (the data for the available side was considered if data for both sides was not available), and the sum of the 3 evaluated joints (range 0–15) was considered.

In the bivariate analysis, the relationship of recurrent falls (outcome variable) with pain and the following variables were evaluated: number of drugs, total strength, balance, functional status, drugs that increase the risk of falling (neuroleptics and hypnotics in this study), affective symptoms, cognitive decline, and visual acuity. The Chi-squared test or Fisher statistical test was used for the categorical independent variables, and the Student T test or Mann-Whitney U test was used for the quantitative variables, according to whether the application criteria were met. The pain variable was treated quantitatively (FPS scale score) and categorically (no pain-mild pain vs moderate-severe pain). This dichotomization was performed taking into account the results of previous studies that emphasize the importance of pain intensity in the analysis of its consequences [10, 13, 32, 33]. The recurrent falls variable was always used as a categorical dichotomous variable.

Following the bivariate analysis, a multivariate model (logistic regression) was developed for each age group considering pain as a quantitative variable (range 0–6 according to FPS score). It included, in addition to age and gender, the variables defined as potential confounding variables and the variables cognitive decline, number of drugs and number of drugs with risk of falls [34]. No covariate was categorized in the models. Each multivariate model was developed in 2 steps: in the first step (step 1), all the aforementioned variables were introduced, and in the second step (step 2), the initial model was simplified to obtain only the significant predictor variables (*p* <  0.05). The pain score was not categorized in the multivariable models to avoid a loss of precision (maximum if dichotomized) in the analyses [35].

Lastly, the possible age (dichotomized in 65–79 years and 80 years and older) and pain (FPS) interaction for recurrent falls was verified through a generalized linear model with the total sample weighted according to gender and age.

In all analyses, a level of statistical significance of 95% (*p* <  0.05) was established, and missing data were excluded from the analyses.

The statistical software SPSS v21.0 and R version 3.5.1 were used.

### Ethical approval and consent to participate

The study was approved by the Ethics Committee for Clinical Research of the Hospital of Mataró-Maresme Health Consortium (Hospital de Mataró-Consorci Sanitari del Maresme) (Acta 10/07) (reference committee of the centre responsible for the study). Written consent was obtained from all participants or their proxy relatives.

## Results

### Sample and monitoring

**Fig. 1:**
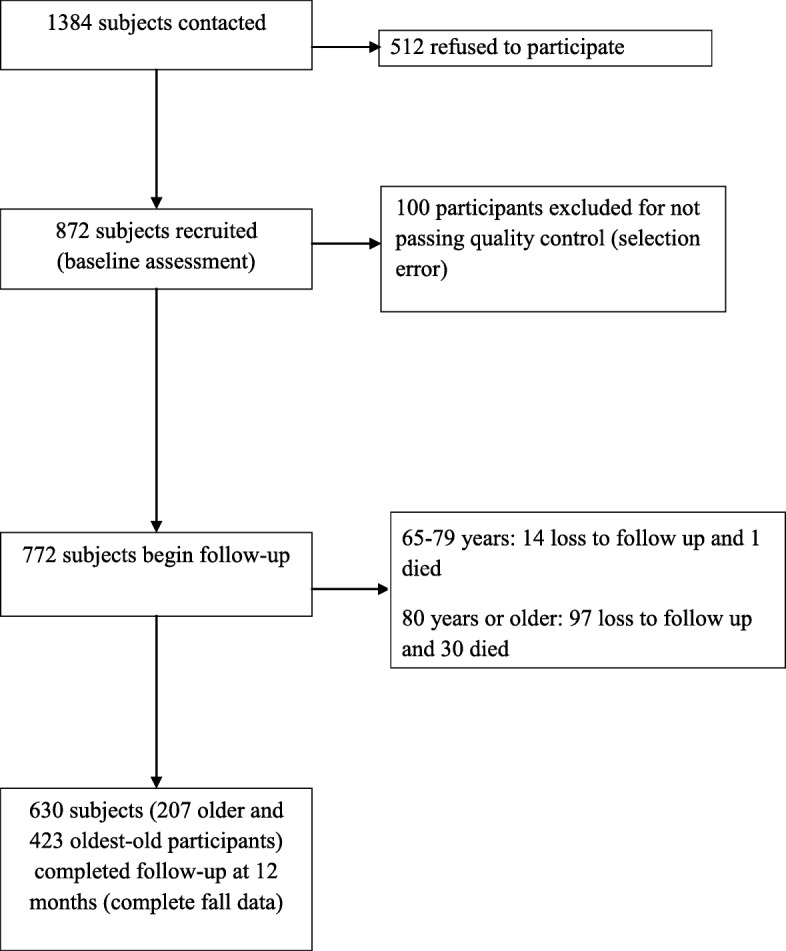
Subject follow-up and loss during the study

Figure 1 (Sample and follow-up) shows the recruitment and follow-up for this study. A total of 222 older participants and 550 oldest-old participants were included. 630 (81.6%) participants (207 older and 423 oldest-old participants) completed the 12-month follow-up, with the mean follow-up time (among the participants who had at least one follow-up control) of 11.85 months (standard deviation [SD] 0.9) for the older group and 11.29 months (SD 2.01) for the oldest-old group. 1 older and 30 oldest-old participants died during the follow-up period

The baseline assessment data according to age group and pain intensity are shown in Table 1. For both age groups, participants with moderate-severe pain were predominantly women, had a higher prevalence of osteoarthritis/arthritis, osteoporosis, and affective symptoms, and consumed a greater number of drugs, drugs with risk of falls and analgesics.

**Table 1:** Baseline assessment at enrolment

Variable	65–79 years			80 or more years		
	no pain - mild pain	moderate-severe pain	p	no pain - mild pain	moderate-severe pain	p
Age (years)						
mean (SD)	72.16 (4.24)	71.54 (4.15)	0.368	84.81 (4.22)	84.67 (4.22)	0.702
minimum-maximum	65–79	65–79		80–99	80–100	
Gender						
women (%)	75 (49.34)	45 (78.95)	< 0.01	187 (58.62)	144 (71.29)	< 0.01
men	77 (50.66)	12 (21.05)		132 (41.38)	58 (28.71)	
Education						
university students (%)	11 (7.28)	3 (5.26)	0.452	18 (5.75)	3 (1.52)	0.038
middle school	21 (13.91)	4 (7.02)		33 (10.54)	14 (7.07)	
elementary school	89 (58.94)	35 (61.40)		175 (55.91)	112 (56.57)	
none	30 (19.87)	15 (26.32)		87 (27.80)	69 (34.85)	
Address						
live alone (nobody) (%)	26 (13.90)	17 (24.64)	0.054	95 (27.30)	52 (22.61)	0.272
partner/spouse	103 (55.08)	29 (42.03)	0.036	114 (32.76)	67 (29.13)	0.507
son/daughter	48 (25.67)	20 (28.96)	0.623	96 (27.59)	73 (31.74)	0.210
grandson/granddaughter	9 (4.81)	3 (4.35)	1.000	24 (6.90)	23 (10)	0.160
non-family caregiver	1 (0.53)	0	1.000	19 (5.46)	15 (6.52)	0.587
Technical assistance for walking at home						
none (%)	137 (95.80)	49 (87.5)	0.614	234 (69.85)	115 (55.29)	< 0.01
furniture support	1 (0.70)	3 (5.36)	0.060	12 (3.58)	12 (5.77)	0.286
person	0	0	1000	9 (2.69)	9 (4.33)	0.334
cane (unipodal/English)/tripod	2 (1.40)	4 (7.14)	0.247	48 (14.33)	40 (19.23)	0.148
walker with/without wheels	1 (0.70)	0	1.000	18 (5.37)	13 (6.25)	0.584
wheelchair	2 (1.40)	0	1.000	14 (4.18)	19 (9.13)	0.027
Technical assistance for walking on the street						
none (%)	130 (91.55)	50 (87.72)	0.648	190 (59.19)	86 (42.16)	< 0.01
furniture support	0	0	1.000	0	2 (0.98)	0.149
person	1 (0.70)	1 (1.75)	0.467	13 (4.05)	14 (6.86)	0.159
cane (unipodal/bipodal)/tripod	9 (6.34)	6 (10.53)	0.767	84 (26.17)	69 (33.82)	0.066
walker with/without wheels	1 (0.70)	0	1.000	12 (3.74)	12 (5.88)	0.235
wheelchair	1 (0.70)	0	1.000	22 (6.85)	21 (10.29)	0.190
Functional status (range 0–6; 0 independent)						
mean (SD)	0.22 (0.63)	0.39 (0.98)	0.292	0.96 (1.74)	1.73 (2.17)	< 0.01
minimum-maximum	0–5	0–6		0–6	0–6	
Body mass index						
mean (SD)	28.84 (4.22)	29.12 (4.6)	0.828	28.07 (4.99)	29.11 (5.21)	0.037
≥ 1 fall in the previous 6 months (%)	32 (21.05)	12 (21.05)	1000	72 (22.64)	79 (39.11)	< 0.01
Chronic diseases						
hypertension (%)	77 (50.66)	33 (57.89)	0.437	152 (47.65)	108 (53.47)	0.209
diabetes mellitus 2 (%)	29 (19.21)	11 (19.30)	1000	50 (15.67)	51 (25.25)	< 0.01
dyslipidaemia (%)	54 (35.76)	26 (45.61)	0.205	61 (19.18)	58 (28.71)	0.014
acute myocardial infarction/other heart diseases (%)	46 (15.18)	28 (24.56)	0.061	87 (13.81)	74 (18.32)	0.134
bronchial asthma/COPD (%)	37 (12.17)	8 (7.02)	0.639	79 (12.42)	52 (12.87)	0.751
arthrosis or arthritis (%)	74 (48.68)	47 (82.46)	< 0.01	169 (52.98)	158 (78.22)	< 0.01
osteoporosis (%)	18 (11.84)	19 (33.33)	< 0.01	35 (11.04)	52 (26)	< 0.01
good visual acuity (%)	134 (88.16)	48 (84.21)	0.489	249 (81.11)	155 (78.68)	0.567
Cognitive decline						
Pfeiffer, 0–2 errors (%)	132 (88.59)	52 (91.23)	0.801	226 (73.14)	142 (70,30)	0.483
Pfeiffer, 3 or more errors	17	5		83	60	
Affective symptoms (Yesavage test: 2 or more points) (%)	21 (13.91)	17 (29.82)	0.015	93 (31.31)	104 (53.06)	< 0.01
Balance (total score), range 0–7						
mean (SD)	6.56 (0.98)	6.49 (0.98)	0.611	5.42 (2.13)	4.59 (2.37)	< 0.01
minimum-maximum	1–7	3–7		0–7	0–7	
Total strength (total score), range 0–34						
mean (SD)	32.69 (2.29)	31.98 (3.88)	0.322	28.94 (8.15)	23.83 (10.59)	< 0.01
minimum-maximum	20–34	12–34		0–34	0–34	
Number of drugs						
mean (SD)	3.56 (2.68)	4.77 (2.85)	< 0.01	4.06 (2.45)	5.06 (2.61)	< 0.01
minimum-maximum	0–12	0–11		0–11	0–12	
1 or more drugs with risk of falls (%)	31 (20.39)	22 (38.60)	< 0.01	93 (29.15)	77 (38.12)	0.029
1 or more analgesics (%)	21 (14.29)	17 (30.36)	< 0.01	81 (25.47)	86 (43.22)	< 0.01

*SD* standard deviation, *IQR* interquartile range, *COPD* chronic obstructive pulmonary disease

### Pain

In the older group, 114 (51.35%) participants reported pain (in all participants, it was possible to assess the presence of pain), of which 43 (37.7%), 42 (36.8%), and 14 (12.3%) reported mild, moderate and severe pain, respectively (in 15 [13.16%] participants, pain intensity could not be evaluated). In the group of oldest-old participants, 286 (52%) participants reported pain (1 participant could not assess the presence of pain); the distribution according to intensity (mild, moderate or severe) was 55 (19.2%), 131 (45.8%), and 69 (24.1%); respectively (in 31 [10.8%] participants, pain intensity could not be evaluated). Among participants with pain, in both the older and oldest-old groups, the most common body locations were the thoracolumbar region (35.1 and 44.7%, respectively) and the lower limbs (34.2 and 54.2%, respectively). The prevalence of pain in the group that did not complete the follow-up was 40% in the older group and 60.5% in the oldest-old group.

### Falls

Regarding falls, 99 falls were registered in the older group and 250 in the oldest-old group. Among participants with data on falls during follow-up, 6.93% (*n* = 12) of the older group and 12.06% (*n* = 51) of the oldest-old group reported 2 or more falls.

### Pain-fall association

The results of the bivariate analysis are shown in Table 2.

**Table 2:** Recurrent falls-pain bivariate analysis

Variables	65–79 years			80 years or older		
	Recurrent faller			Recurrent faller		
	yes	no	p	yes	no	p
Categorical	n (%)	n (%)		n (%)	n (%)	
Moderate-severe pain	8 (61.54)	46 (25.70)	< 0.01	24 (48.0)	122 (34.56)	0.064
Poor visual acuity	3 (21.43)	27 (14.29)	0.467	12 (23.53)	63 (17.70)	0.315
Quantitative	mean (SD)	mean (SD)	p	mean (SD)	mean (SD)	p
Pain (FPS, range 0–6)	2.85 (1.73)	1.34 (1.69)	0.002	2.04 (2.06)	1.58 (1.97)	0.125
Number of drugs that increase the risk of falling	1.07 (0.92)	0.33 (0.62)	< 0.01	0.53 (0.75)	0.47 (0.74)	0.561
Affective symptoms (Yesavage score)	2.00 (2.00)	0.72 (1.10)	0.034	1.96 (1.63)	1.28 (1.48)	0.003
Cognitive decline (Pfeiffer score)	1.36 (1.50)	0.95 (1.16)	0.214	1.67 (2.42)	1.72 (2.24)	0.868
Number of drugs	5.21 (2.72)	3.88 (2.71)	0.077	5.87 (2.78)	4.28 (2.54)	< 0.01
Muscle strength, range 0–15	13.79 (1.63)	14.39 (1.13)	0.194	11.94 (4.32)	12.55 (3.68)	0.284
Balance, range 0–7	5.38 (1.56)	6.63 (0.84)	0.014	4.84 (2.34)	5.36 (2.08)	0.127
Functional situation (Katz, range 0–6)	0.64 (1.01)	0.23 (0.60)	0.156	1.51 (1.97)	1.09 (1.81)	0.421

*FPS* Face Pain Scale

In the older group, 3.6% of participants with no pain-mild pain and 14.8% of participants with moderate-severe pain presented recurrent falls. In the oldest-old group, recurrent falls occurred in 10.1 and 16.4% of participants with no pain-mild pain and moderate-severe pain, respectively. Thus, moderate to severe pain was associated with an increased risk of recurrent falls in the older group (RR [95% CI]: 3.57 [1.15–9.11]) but not in the oldest-old group (RR: 1.62 [0.97–2.72]). Pain intensity was higher in the group of participants with recurrent falls; however, the difference was significant only in the older group (FPS score 2.85 vs. 1.34, *p* = 0.002).

The results of the multivariate analysis are shown in Table 3. In the older group, pain was associated with recurrent falls, with an associated odds ratio (OR) of 1.47 (95% CI 1.08–2.00; beta 0.3864) for each unit increase in pain intensity (participants with the most severe pain [FPS 6] had OR 10.16 regarding to participants without pain [FPS 0]). In the oldest-old group, pain was not associated with recurrent falls.

**Table 3:** Recurrent pain-fall multivariate analysis

Variable^a^	65–79 years (*n* = 192)			80 years or more (*n* = 402)		
	OR	CI 95%	p	OR	CI 95%	p
Pain	1.47	1.08–2.00	0.017			
Drugs with risk of falls	3.56	1.75–7.25	< 0.01	0.55	0.33–0.90	0.018
Balance	0.45	0.28–0.73	< 0.01			
Number of drugs				1.26	1.11–1.43	< 0.01
Age				1.09	1.02–1.15	< 0.009
Affective symptoms				1.28	1.06–1.56	0.012

(^a^): The initial variables (step 1) of the model for both age groups were pain, muscle strength, drugs that increase the risk of falls, balance, cognitive decline, age, gender, affective symptoms and number of drugs

Given the negative result for a pain-recurrent falls association and loss to follow-up in the oldest-old group, we calculated the a posteriori statistical power (type II error) of this result. The calculated power was 50% for the bivariate analysis of moderate-severe pain (unadjusted) vs. recurrent falls; and we calculated that our sample had a statistical power of 90% to find a proportions difference of 12.11% (proportions difference in our sample was 6% [10.1 vs 16.4%]).

Regarding the weighted analysis of a possible interaction between age (dichotomized) and pain over recurrent falls, the estimated parameter for the interaction (beta = 2.56) was not significant (*p* = 0.129).

## Discussion

This study confirms the independent relationship of pain with recurrent falls in the older people. Various mediators have been proposed regarding the relationship of pain with recurrent falls: impaired balance, muscle weakness, and depression [10, 13]. However, even controlling for these factors in the multivariate analysis, pain persisted as an independent risk factor; therefore, it could be assumed that other mediators play a prominent role. In this sense, it is worth noting the known role of cognition [14, 36], especially with regard to impairment in attention and executive function [37–39]. Although our multivariable models included cognitive assessment, cognition was evaluated through the Pfeiffer test [24, 25], which is not specifically designed for the detection of attentional or executive profile failures.

We did not find a relationship between pain and recurrent falls in the oldest-old population. However, this result is inconclusive due to the lack of sufficient statistical power. We believe that the rate of loss to follow-up and the smaller difference between participants with falls and without falls according to pain intensity in this group (10.1 vs 16.4%) were the main factors determining the low power achieved. Anyway our sample had enough statistical power to exclude a difference proportions longer than 12.11% in this age group. Of the prospective studies performed previously [10–15], only the studies by Marshall et al. [14, 15], who evaluated thoracolumbar pain as a risk factor for recurrent falls, analysed this association in a differentiated manner according to age group and found that the relationship was positive only in the group < 75 years, in both men and women. Although our results and those previously mentioned [14, 15] cannot exclude chance, our summation results reinforce the hypothesis that the recurrent pain-fall relationship in this age group is at least less intense or that other risk factors are more prominent with respect to the group of 65–79 years older people. Surprisingly, in the oldest-old group, drugs that increase the risk of falling paradoxically showed a protective effect but we think this is because a residual confounding effect more than a real phenomenon.

In addition to these results, interestingly, the multivariate analysis for the oldest-old group did not find that classic risk factors for falls (drugs that increase the risk of falling or balance), increased the risk of falling. Beside this, the quantitative differences between participants with falls and without falls with respect to risk factors (Table 2) were much lower in the oldest-old group than in the older group. If our results are confirmed in other samples, it would be important to investigate whether the risk factors for falls known to date act differently in the oldest-old population or if certain risk factors have more impact in this age group. In this way we point out cardiovascular changes and syncope because their known clinical impact on mortality and functional deterioration, specially in the oldest-old population [40, 41]. Unfortunately we could not evaluate this topic in our sample.

A broad spectrum of the older and oldest-old community population was included. Our results cannot be extrapolated to the institutionalized or hospitalized population. We chose a cut-off point of 80 for the separation of age groups because it is the usual cut-off point considered in the literature.

Although loss to follow-up was significant, the prevalence of pain among those who did not complete follow-up showed no significant differences compared to those who completed follow-up (51% vs. 40% in the older group, and 52% vs. 60.5% in the oldest-old group). Although this does not exclude the appearance of bias, we do not believe that the effect was important in our results. We did not control the pain variable during follow-up; therefore, some participants could have been classified in the wrong group if the absence or presence of pain changed during follow-up. However, we believe that this bias would have little impact on our results (the study by Munch et al. [13] found no evidence of relevant influence on the results of a recurrent pain-fall association with a change in pain status during follow-up). Lastly, we did not differentiate participants with transient post-fall pain in the baseline assessment; therefore, we recognize, in addition to the invalidity of the cause-effect sequence in these cases, the possibility of bidirectional bias: overestimation of risk if falls continued to occur during follow-up and underestimation if there were no falls.

## Conclusions

In conclusion, potentially modifiable and highly prevalent pain is a risk factor for recurrent falls in the older population (65–79 years). We have not been able to demonstrate that this relationship is maintained in the oldest-old population (80 or more years).

## Data Availability

The datasets used and/or analysed during the current study are available from the corresponding author on reasonable request.

## Abbreviations

CI: Confidence interval
FPS: Face Pain Scale
OR: Odds ratio
RR: Relative risk
SD: Standard deviation
